# Battle for the thermostat: Gender and the effect of temperature on cognitive performance

**DOI:** 10.1371/journal.pone.0216362

**Published:** 2019-05-22

**Authors:** Tom Y. Chang, Agne Kajackaite

**Affiliations:** 1 USC Marshall School of Business, Los Angeles, CA, United States of America; 2 WZB Berlin Social Science Center, Berlin, Germany; Middlesex University, UNITED KINGDOM

## Abstract

This paper studies differences in the effect of temperature on cognitive performance by gender in a large controlled lab experiment (N = 543). We study performance in math, verbal and cognitive reflection tasks and find that the effects of temperature vary significantly across men and women. At higher temperatures, women perform better on a math and verbal task while the reverse effect is observed for men. The increase in female performance in response to higher temperature is significantly larger and more precisely estimated than the corresponding decrease in male performance. In contrast to math and verbal tasks, temperature has no impact on a measure of cognitive reflection for either gender. Our findings suggest that gender mixed workplaces may be able to increase productivity by setting the thermostat higher than current standards.

## Introduction

The fact that women generally prefer higher indoor temperatures than men is well supported by survey evidence [1–4]. This difference in preferences, sometimes referred to as the “battle of the thermostat,” is passionately discussed in popular culture and has received considerable media attention [5–7]. Surprisingly then, the research examining the impact of temperature on cognitive performance [8] has not explored a link between gender and temperature response.

To address this gap we ran a large laboratory experiment in which over 500 individuals were asked to perform a set of cognitive tasks (math, verbal and cognitive reflection), subject to experimentally manipulated indoor temperatures. We find that, for math and verbal tasks, consistent with their subjective temperature preferences, women perform better both on the extensive and intensive margins at high temperatures than at low temperatures (i.e., women both attempt to solve, and correctly solve more math and verbal tasks at higher vs. lower temperatures). Men display the opposite pattern, performing better at lower temperatures. In contrast to math and verbal tasks, temperature has no impact on cognitive reflection test performance for either gender.

Overall, our results suggest that gender is an important factor not only in determining the impact of temperature on comfort but also on productivity and cognitive performance. Additionally, given the lack of attention to gender in mediating the impact of temperature on performance, our results may explain in part the inconsistent results of previous studies on the relationship between temperature and cognitive performance [9–11].

## Experimental design

To study the effect of temperature on performance, we conducted a laboratory experiment with 543 students in Berlin, Germany (see Materials and Methods and Supporting Information (SI) for instructions and details on procedure). We used a between subject design by varying the temperature from 16.19 to 32.57°C between sessions. In each session, participants were given the same set of tasks which were monetarily incentivized based on performance. These tasks were:

### Adding up numbers task (Math)

In this task, participants were asked to add up five two-digit numbers without using a calculator (the task is equivalent to the task used by M. Niederle and L. Vesterlund [12]). There were 50 problems and participants had 5 minutes to work on them. Participants could complete as many problems as they wanted in the available time, and were rewarded only for correct answers.

### Words building task (Verbal)

Participants were provided with a set of ten letters: ADEHINRSTU. They were asked to build as many German words as possible in 5 minutes (the task was equivalent to the one introduced by Eckartz et al. [13]). Participants were rewarded for each valid word they submitted, with the payment increasing over-proportionally with the length of the word.

### Cognitive reflection test (CRT)

Participants had 5 minutes to answer three original CRT questions introduced by S. Frederick [14]. For example, the first question was: “A bat and a ball cost 1.10 EUR in total. The bat costs 1.00 EUR more than the ball. How much does the ball cost?” In a cognitive reflection test, the questions are such that the intuitive answer is the wrong answer. In this case, the intuitive answer is that the ball costs 10 cents. This test has been extensively used in the psychology literature and is highly correlated with various measures of mental heuristics and biases and measures of cognitive ability (e.g., IQ) [15].

## Econometric model

We use ordinary least squares (OLS) regressions to map the relationship between temperature and performance. Our basic linear regression is given by the following equation:

$$Y_{ij}=\alpha +\beta {Temp}_{j}+X_{ij}\gamma +\epsilon_{ij}$$ (1)

where *i* references an individual, j references an experimental session, *Temp_j_* is the temperature in the room during session j, and *X_ij_* is a vector of the observable characteristics of the individual and session that might influence performance. The dependent variable *Y_ij_* is a measure of individual i’s performance on the math task, verbal task, CRT, and a measure of the total number of answers attempted (since all subjects attempted to answer all three CRT questions, this is a normalized, equally weighted measure of the number of math questions attempted and the number of words submitted).

An alternative estimation strategy involves a non-linear identification of the temperature-performance relationship. Specifically, we replace *Temp_j_* with a vector of temperature dummies corresponding to the following temperature bins: less than 20, 20–25, 25–30, and more than 30 degrees Celsius.

Finally, to estimate the role gender plays in the relationship between temperature and performance, we interact our linear and non-linear measures of temperature with a male dummy.

## Empirical results

### Assessing the validity of the random assignment

The temperature was randomly assigned across sessions. The participants were recruited via an online recruiting system and were randomly assigned to sessions (see Materials and Methods). To see whether randomization worked, we analyze the relationship between individual subject characteristics and temperature, both measured linearly and non-linearly. Across both specifications, we find no statistically meaningful difference in observable subject characteristics (see S1 Table), which suggests randomization was successful.

### Estimates of the effect of temperature on cognitive performance

**Fig 1:**
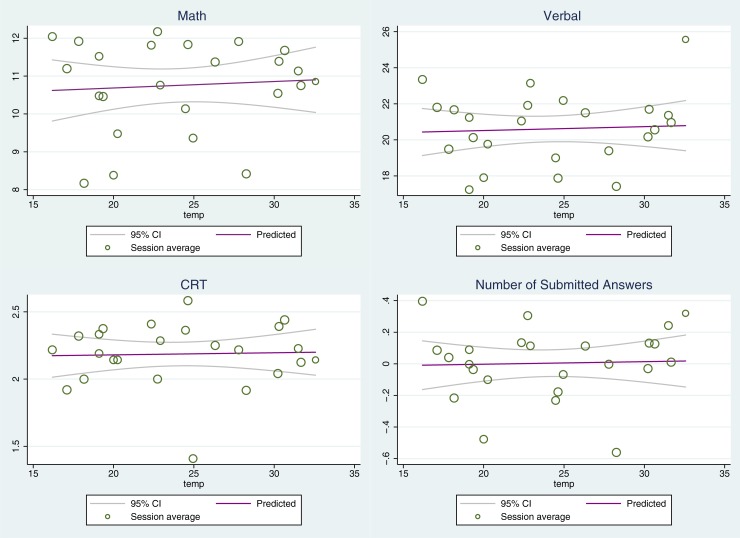
The relationship between temperature and performance. This figure presents estimates of the impact of temperature on performance (math, verbal, CRT and normalized measure of attempted answers). The circles represent the average performance and temperature for each experimental session, with circle size proportional to the number of subjects. The plotted solid line gives the linear projection of outcomes, with the dashed lines presenting the 95% confidence interval around the solid line.

Fig 1 shows the relationship between temperature and performance across the three tasks and the normalized measure of attempted answers. From Fig 1 there does not appear to be a meaningful relationship between temperature and performance in any of the performance measures.

Table 1 statistically summarizes the graphical findings in Fig 1 by reporting estimates (and standard errors) from regressions. Since our prior tests show that our samples are balanced across treatment conditions, the estimates presented here do not include any individual level controls. Nonetheless, our estimates are robust to the inclusion of our full set of observable characteristics. Consistent with Fig 1, the regression results show no statistically significant relationship between performance and temperature across both linear and non-linear specifications.

**Table 1:** OLS estimates of the impact of temperature on performance.

	(1)	(2)	(3)	(4)	(5)	(6)	(7)	(8)
	Math		Verbal		CRT		Total Attempted	
*Temperature*	0.0248		0.018		0.0037		0.0022	
	[0.0429]		[0.0717]		[0.0065]		[0.0088]	
*Temperature < 20*		0.0987		0.4876		-0.0333		0.0956
		[0.6039]		[0.9285]		[0.1173]		[0.1030]
*Temperature 25 to 30*		-0.209		-0.796		-0.1065		-0.1085
		[1.0822]		[1.1919]		[0.1563]		[0.2022]
*Temperature > 30*		0.5085		1.1042		0.0551		0.1754+
		[0.4576]		[0.8397]		[0.1188]		[0.0976]
*Mean Value*	10.75		20.6		2.19		0	
*R-squared*	0.0423	0.0438	0.0037	0.0086	0.0832	0.0851	0.0061	0.0145
*Observations*	542	542	542	542	542	542	542	542

The table presents OLS regression results. Column 1, 3, 5 and 7 use linear temperature as the independent variable. Columns 2, 4, 6, and 8 use temperature bin dummies (less than 20, 25–30, and more than 30 degrees Celsius) with the range of 20–25 degrees Celsius as the reference group. Standard errors in brackets are clustered on experimental session. A plus sign by an estimate indicates statistical significance at the 10-percent level, one asterisk at the 5-percent level, and two asterisks at the 1-percent level.

### Estimates of the effect of temperature on cognitive performance by gender

**Fig 2:**
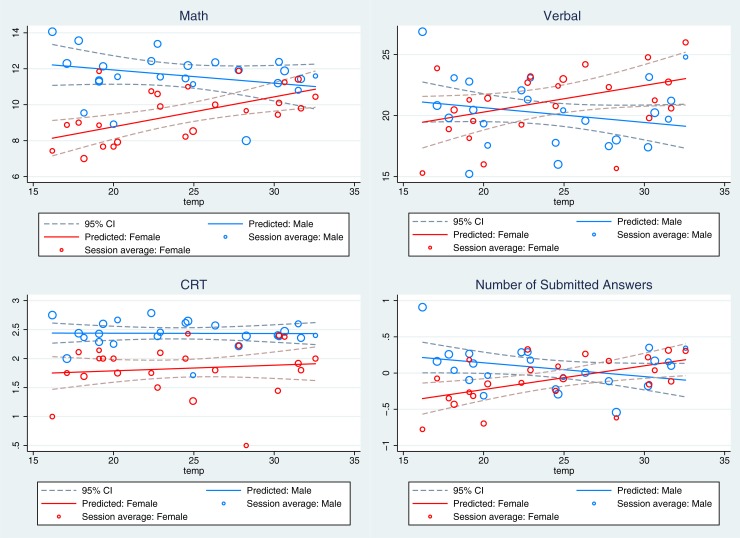
The relationship between temperature and performance by gender. This figure presents estimates of the impact of temperature on performance (math, verbal, CRT and normalized measure of attempted answers) separately for males and females. The circles represent the average performance and temperature for each experimental session divided by gender, with circle size proportional to the number of subjects. The plotted solid line gives the linear projection of outcomes, with the dashed lines presenting the 95% confidence interval around the solid line.

Fig 2 duplicates Fig 1, but breaks down the relationship by gender. In line with Fig 1, Fig 2 shows no relationship between temperature and CRT scores. However, unlike Fig 1, Fig 2 shows a meaningful relationship between temperature and performance for math and verbal tasks. The relationship between performance in math and verbal tasks and temperature is different for males and females, with males performing best at lower temperatures and females performing best at higher temperatures. The lack of a relationship in the full sample described above masked a heterogeneous effect of temperature on performance by gender.

Table 2 presents a more formal test of the visual differences in Fig 2, reporting estimates from the regression equations specified above. The estimates presented here include only a control for gender, but are robust to the inclusion of the full set of observable characteristics. Consistent with Fig 2, we see that for the math task (Columns 1 and 2), temperature is associated with an increase in the performance of women and a decrease in the performance of men. Across specifications, both the increase in female performance and the differential effect of temperature on males relative to females are economically meaningful and statistically significant. The coefficients from the linear specification indicate that a one-degree Celsius increase in ambient temperature is associated with 0.17 (1.76%) increase in the number of math questions correctly answered by women (p-value < 0.001). In contrast, the implied decrease in male performance is generally small and statistically insignificant. Men submit 0.07 (0.63%) fewer correct answers when temperature is increased by one degree (p-value = 0.205). To put the magnitude of these effects in perspective, the well-known, long-standing gap in performance between high school boys and girls on the math portion of the SAT is approximately 4% [16].

**Table 2:** OLS estimates of the impact of temperature on performance by gender.

	(1)	(2)	(3)	(4)	(5)	(6)	(7)	(8)
	Math		Verbal		CRT		Total Attempted	
*Temperature*	0.1666**		0.2182*		0.0098		0.0328**	
	[0.0416]		[0.0980]		[0.0138]		[0.0094]	
Male * Temperature	-0.2402**		-0.3392+		-0.0103		-0.0518*	
	[0.0648]		[0.1847]		[0.0182]		[0.0192]	
*Temperature < 20*		-0.6917		-1.5208		0.0292		-0.2016
		[0.7121]		[1.1642]		[0.1816]		[0.1315]
*Temperature 25 to 30*		1.4250+		0.1925		-0.1475		0.1189
		[0.7297]		[2.0109]		[0.3841]		[0.2162]
*Temperature > 30*		1.2560*		1.1953		0.1953		0.2069
		[0.5377]		[1.2063]		[0.1867]		[0.1259]
Male * Temperature < 20		1.2095		3.2801		-0.1110		0.4819*
		[0.7873]		[2.2658]		[0.2073]		[0.2200]
Male * Temperature 25 to 30		-2.5699*		-1.4081		0.0492		-0.3375**
		[1.1799]		[1.9977]		[0.4039]		[0.1019]
Male * Temperature > 30		-1.3416*		-0.1423		-0.2524		-0.0538
		[0.5091]		[1.9222]		[0.1675]		[0.1607]
*Male*	7.9673**	2.4917**	7.1437	-1.7458	0.8547+	0.7021**	1.4062**	0.0757
	[1.5931]	[0.3186]	[4.7284]	[1.1962]	[0.4594]	[0.1052]	[0.49]	[0.0908]
*Mean Value*	10.75		20.6		2.19		0	
*R-squared*	0.0557	0.0579	0.0139	0.0181	0.0838	0.0877	0.0232	0.0326
*Observations*	542	542	542	542	542	542	542	542

The table presents OLS regression results. Column 1, 3, 5 and 7 use linear temperature as the independent variable. Columns 2, 4, 6, and 8 use temperature bin dummies (less than 20, 25–30, and more than 30 degrees Celsius) with the range of 20–25 degrees Celsius as the reference group. Standard errors in brackets are clustered on experimental session. A plus sign by an estimate indicates statistical significance at the 10-percent level, one asterisk at the 5-percent level, and two asterisks at the 1-percent level.

For the verbal task (Columns 3 and 4), the coefficients for both the linear and non-linear specifications follow the same general pattern as for the math task, but are less precise. The coefficients from the linear regression estimate imply that a temperature increase of one degree Celsius increases female performance on the verbal task by 1.03% (p-value = 0.036) and decreases male performance by 0.6% (p-value = 0.331) The difference in effects between men and women is not statistically significant (p-value = 0.079).

In contrast to the math and verbal tasks, and consistent with Fig 2, we find that temperature has no significant impact on CRT scores for either men or women (Columns 5 and 6).

Finally, Columns 7 and 8 examine the effect of temperature on the number of answers attempted for the math and verbal tasks. Similar to the number of correct answers, we find that temperature is positively correlated with the number of answers attempted by female participants and negatively correlated with the number of answers attempted by male participants. The regression coefficients imply that a one-degree Celsius increase in temperature leads to a 0.033 standard deviation increase in the number of answers attempted by females subjects (p-value = 0.002), and a 0.019 standard deviation decrease in the number of questions attempted by male subjects (p = 0.187). The difference between the two effects is significant with p-value = 0.013.

Importantly, the increase in the number of submitted answers does not lead to higher error rates. Indeed, for women, higher temperatures are associated with lower error rates, although the results are only marginally statistically significant (see S2 Table).

## Discussion

Taken together, these results show that within a temperature range of 16 and 33 degrees Celsius, females generally exhibit better cognitive performance at the warmer end of the temperature distribution while men do better at colder temperatures. The increase in female cognitive performance appears to be driven largely by an increase in the number of submitted answers. We interpret this as evidence that the increased performance is driven in part by an increase in effort. Similarly, the decrease in male cognitive performance is partially driven by a decrease in observable effort. Importantly, the increase in female cognitive performance is larger and more precisely estimated than the decrease in male performance.

That these results were completely masked when differential-by-gender effects were not taken into account could be one possible explanation for the inconsistent results found in the prior literature on temperature and cognitive performance. At the very least, it suggests that future studies on the effect of temperature should examine potential heterogeneous effects across gender.

Interestingly, we find no effect of temperature on CRT scores overall, or by gender. Given the relationship between cognitive reflection and violent crime [17] and the well-documented relationship between temperature and violence [18–20], this result was surprising. One potential, albeit purely speculative, explanation is that the more behavioral effects of temperature, such as effects on reflection or aggression, manifest at longer time horizons than a one-hour experimental session. Another is that the effect of temperature on violent tendencies operates through a channel unrelated to cognitive reflection.

Our results contribute to the growing experimental economics and psychology literature on gender differences. Recent experimental evidence suggests that women are less competitive [21, 22], more risk-averse [23, 24] and more honest [25, 26] than men. There is also some evidence showing that women are more altruistic [27, 28], more cooperative [29] and more emotive in moral dilemma judgments [30] than men.

Methodologically, our results call for some caution for laboratory experiments in psychology, economics and other social sciences with respect to gender composition in experimental sessions. Since we show that ordinary variations in room temperature can affect cognitive performance significantly and differently for men and women, experimental social scientists should take this into account when both designing experiments and interpreting the results.

Ultimately, our results potentially raise the stakes for the battle of the thermostat, suggesting that it is not just about comfort, but also about cognitive performance and productivity. Given the relative effect sizes, our results suggest that in gender-balanced workplaces, temperatures should be set significantly higher than current standards.

## Materials and methods

This study was conducted with an IRB approval from University of Southern California and with an approval of an ethics committee at WZB Berlin Social Science Center. We recruited students in Berlin, Germany via the online recruiting system for economic experiments ORSEE [31]. The participants were randomly selected from the subject pool in ORSEE and none of them participated in more than one session. We excluded participants who have previously participated in ten or more experiments in this laboratory.

Overall, 543 students participated in our experiment. One participant did not complete the post-questionnaire and due to missing demographics we exclude this participant of our analyses. Including the participant does not change the quantitative results. Out of the remaining 542 participants, 41.14% were female. Table 3 summarizes the demographics of our sample. Our sample consisted exclusively out of students from universities in Berlin. The advantages of this subject pool is that they are relatively easy to recruit and homogenous in their cognitive skills. The disadvantage of this subject pool is that it is not representative of the whole population with respect to age and education level.

**Table 3:** Demographic details of the participants.

	Mean	SD	Median	Min	Max
Male	0.59	0.49	1	0	1
Age	24.06	4.87	23	17	55
Major in Economics/Business	0.38	0.49	0	0	1
Native in German	0.79	0.41	1	0	1

We conducted the experiment at the experimental economics laboratory at Technical University Berlin. The sessions took place from 25^th^ of September until 6^th^ of December 2017. In total, we conducted 24 sessions with 23–25 participants a session, depending on the show-up rates.

After arrival in the laboratory and before entering the main laboratory room where the experiment takes place, participants picked a random number with a seat assignment and were informed by the experimenter that this experiment includes temperature variations. The participants were also told that if they do not want to stay in the experiment, they can leave right now or at any time during the experimental session and that they will be paid a show-up fee in this case. Note that we did not obtain informed consent of the participants, since the need for consent was waived by the ethics committee. After entering the laboratory room and taking their seat, the experimenter informed the participants about general rules of the laboratory and handed out instructions. Participants were told that there are several parts of the experiment and that they will be informed about the content of the next task only when the current task is finished. The tasks were, in order: a cognitive reflection test, an adding up numbers task, and a words building task (see *SI* for instructions of the experiment with exact descriptions of the tasks). After completing the tasks, participants were asked to fill in a post-questionnaire including demographic questions and questions regarding the motives behind the decisions in the experiment. Then the experimenters collected instructions with participants’ answers, checked the responses for correctness and calculated payoffs. At the end, participants privately received their payoffs in cash and left the laboratory. On average, an experimental session lasted one hour.

In all experimental sessions, participants worked on the same tasks. However, the temperature of the laboratory differed between the sessions. The average temperature in a session ranged between 16.19 and 32.57 Celsius, which we varied using AC and electronic heaters. The participants were told before entering the laboratory that the experiment is about temperature and that they are allowed to leave the laboratory at any time. No participant left the laboratory before or during the session. Table 4 displays temperature variations in the experiment.

**Table 4:** Temperature overview in the experiment.

Temperature group	Temperature range (N)	Average temperature
Temperature < 20	16.19–19.35°C (164)	18.09°C
Temperature 20 to 25	20.00–24.95°C (176)	22.84°C
Temperature 25 to 30	26.33–28.26°C (71)	27.46°C
Temperature > 30	30.23–32.57°C (132)	31.04°C

The table displays temperature ranges, average temperatures and the number of observations in each temperature category.

## Supporting information

S1 Table Probit and OLS estimates of subject characteristics and temperature. (DOCX)

S2 Table OLS estimates of the impact of temperature on error rates by gender. (DOCX)

S3 Table Experimental data. (DTA)

S1 Text Instructions of the experiment. (DOCX)
