# Supplementary material for: Battle for the thermostat: Gender and the effect of temperature on cognitive performance
Source: PLoS One. 2019 May 22;14(5):e0216362. doi: 10.1371/journal.pone.0216362 (PMC6530830; doi:10.1371/journal.pone.0216362)
Supplement: S1 Table — (DOCX) [file pone.0216362.s001.docx]

**S1 Table. Probit and OLS estimates of subject characteristics and temperature**

|  | (1) | (2) | (3) | (4) | (5) | (6) | (7) | (8) | (9) | (10) | (11) | (12) | |
| --- | --- | --- | --- | --- | --- | --- | --- | --- | --- | --- | --- | --- | --- |
|  |  |  |  |  |  |  |  |  |  |  |  |  | |
|  | Gender (male=1) | | German | | Econ Major | | Age | | Enjoys Math | | Enjoys Words | |  |
|  |  |  |  |  |  |  |  |  |  |  |  |  | |
| *Temperature* | -0.009 |  | -0.0066 |  | -0.006 |  | 0.0103 |  | 0.0148 |  | 0.0106 |  | |
|  | [0.0106] |  | [0.0116] |  | [0.0108] |  | [0.0398] |  | [0.0237] |  | [0.0199] |  | |
|  |  |  |  |  |  |  |  |  |  |  |  |  | |
| *Temperature < 20* |  | 0.2227 |  | 0.2256 |  | 0.2164 |  | -0.5779 |  | -0.0391 |  | 0.1452 | |
|  |  | [0.1380] |  | [0.1536] |  | [0.1391] |  | [0.5284] |  | [0.3082] |  | [0.2553] | |
|  |  |  |  |  |  |  |  |  |  |  |  |  | |
| *Temperature 25 to 30* |  | 0.2654 |  | 0.2836 |  | 0.1576 |  | -0.4116 |  | -0.3378 |  | 0.3291 | |
|  |  | [0.1798] |  | [0.2043] |  | [0.1797] |  | [0.7373] |  | [0.4042] |  | [0.3081] | |
|  |  |  |  |  |  |  |  |  |  |  |  |  | |
| *Temperature > 30* |  | 0.0383 |  | 0.0734 |  | 0.1169 |  | -0.3466 |  | 0.3087 |  | 0.2292 | |
|  |  | [0.1449] |  | [0.1588] |  | [0.1480] |  | [0.5763] |  | [0.3209] |  | [0.2725] | |
|  |  |  |  |  |  |  |  |  |  |  |  |  | |
| *Mean Value* | 0.59 | | 0.79 | | 0.38 | | 24.06 | | 5.99 | | 7.19 | |  |
|  |  |  |  |  |  |  |  |  |  |  |  |  | |
| *R-squared* | 0.0010 | 0.0056 | 0.0006 | 0.0058 | 0.0004 | 0.0035 | 0.0001 | 0.0023 | 0.0007 | 0.0048 | 0.0005 | 0.0024 | |
| *Observations* | 542 | 542 | 542 | 542 | 542 | 542 | 542 | 542 | 542 | 542 | 542 | 542 | |
|  |  |  |  |  |  |  |  |  |  |  |  |  | |
| The table presents Probit and OLS regression results. Columns 1-6 present the results of a Probit model, while columns 7-12 present the results of an OLS regression. Odd numbered columns use linear temperature; in even numbered columns, temperature bins are used with the range of 20-25 degrees Celsius the reference group. Pseudo R-squared values are provided for the Probit regressions. "German" indicates whether the subject is a native German speaker. "Enjoys Math" and "Enjoys Words" are self-reported measures that can take integer values of 0 to 10. Mean Value is the mean value of the dependent variable. In brackets are robust standard errors. A plus sign by an estimate indicates statistical significance at the 10-percent level, one asterisk at the 5-percent level, and two asterisks at the 1-percent level. | | | | | | | | | | | | |  |
