# Supplementary material for: Battle for the thermostat: Gender and the effect of temperature on cognitive performance
Source: PLoS One. 2019 May 22;14(5):e0216362. doi: 10.1371/journal.pone.0216362 (PMC6530830; doi:10.1371/journal.pone.0216362)
Supplement: S2 Table — (DOCX) [file pone.0216362.s002.docx]

**S2 Table. OLS estimates of the impact of temperature on error rates by gender**

|  |  | (1) | (2) |  |
| --- | --- | --- | --- | --- |
|  |  |  |  |  |
|  |  | Error Rate | |  |
|  |  |  |  |  |
|  | *Temperature* | -0.0017+ |  |  |
|  |  | [0.0009] |  |  |
|  |  |  |  |  |
|  | *Male * Temperature* | 0.0012 |  |  |
|  |  | [0.0012] |  |  |
|  |  |  |  |  |
|  | *Temperature < 20* |  | 0.0144 |  |
|  |  |  | [0.0135] |  |
|  |  |  |  |  |
|  | *Temperature 25 to 30* |  | -0.0234 |  |
|  |  |  | [0.0180] |  |
|  |  |  |  |  |
|  | *Temperature > 30* |  | -0.0040 |  |
|  |  |  | [0.0105] |  |
|  |  |  |  |  |
|  | *Male * Temperature < 20* |  | 0.0110 |  |
|  |  |  | [0.0175] |  |
|  |  |  |  |  |
|  | *Male * Temperature 25 to 30* |  | 0.0375 |  |
|  |  |  | [0.0288] |  |
|  |  |  |  |  |
|  | *Male * Temperature > 30* |  | 0.0206 |  |
|  |  |  | [0.0133] |  |
|  |  |  |  |  |
|  | *Male* | -0.0438 | -0.0293** |  |
|  |  | [0.0300] | [0.0099] |  |
|  |  |  |  |  |
|  | *R-squared* | 0.0098 | 0.0171 |  |
|  | *Observations* | 542 | 542 |  |
|  |  |  |  |  |
| The table presents OLS regression results. Column 1uses linear temperature as the independent variable; Column uses temperature bin dummies (less than 20, 25-30, and more than 30 degrees Celsius) with the range of 20-25 degrees Celsius as the reference group. Standard errors in brackets are clustered on experimental session. A plus sign by an estimate indicates statistical significance at the 10-percent level, one asterisk at the 5-percent level, and two asterisks at the 1-percent level. | | | | |
