# Supplementary material for: Battle for the thermostat: Gender and the effect of temperature on cognitive performance
Source: PLoS One. 2019 May 22;14(5):e0216362. doi: 10.1371/journal.pone.0216362 (PMC6530830; doi:10.1371/journal.pone.0216362)
Supplement: S1 Text — (DOCX) [file pone.0216362.s004.docx]

# S3 Instructions of the experiment

Original instructions were in German.

**Instructions**

Welcome to our experiment. Please read the instruction carefully. If you have a question, please raise your hand and we will come over to you. Please do not communicate with other participants during the experiment.

This experiment is about temperature.

Your participation is voluntary. You are allowed to leave the room at any time.

Every participant will receive 3EUR for attending, which will be paid out independently of the decisions made in the experiment.

Furthermore, you will be able to earn additional money. At the end of the experiment, you will receive the income you earned over the course of the experiment plus the 3EUR for attending in cash.

Your decisions are private and no other participant will know about them.

The experiment will consist of several parts and a post-questionnaire. You will receive instructions for each part of the experiment before the part of the experiment starts.

Do you have any questions?

**Part 1**

Below are several problems that vary in difficulty. Try to answer as many as you can in 5 minutes. For each question you answer correctly, you will receive 1EUR.

A bat and a ball cost 1.10EUR in total. The bat costs 1.00EUR more than the ball. How much does the ball cost? _____

If it takes 5 machines 5 minutes to make 5 widgets, how long would it take 100 machines to make 100 widgets? _____

In a lake, there is a patch of lily pads. Every day, the patch doubles in size. If it takes 48 days for the patch to cover the entire lake, how long would it take for the patch to cover half of the lake? ______

**Part 2**

Your task is to add up five two-digit numbers (see the example below). Solve as many problems as you can. You will have 5 minutes from the time the experimenter tells you to start. You may use scratch paper, but use of a calculator is not allowed.

You will receive 0.10EUR per correct answer.

| EXAMPLE | |  |  |  | ANSWER |
| --- | --- | --- | --- | --- | --- |
| 88 | 21 | 79 | 78 | 16 | 282 |
| QUESTIONS | |  |  |  |  |
| 18 | 76 | 37 | 51 | 23 |  |
| 73 | 70 | 27 | 50 | 35 |  |
| 43 | 69 | 31 | 71 | 96 |  |
| 63 | 79 | 48 | 12 | 13 |  |
| 28 | 23 | 71 | 28 | 52 |  |
| 31 | 64 | 48 | 48 | 45 |  |
| 22 | 61 | 55 | 18 | 35 |  |
| 53 | 23 | 16 | 40 | 75 |  |
| 24 | 15 | 48 | 50 | 21 |  |
| 27 | 36 | 25 | 76 | 25 |  |
| 42 | 43 | 43 | 17 | 10 |  |
| 86 | 46 | 50 | 54 | 31 |  |
| 53 | 51 | 94 | 97 | 58 |  |
| 22 | 98 | 87 | 77 | 56 |  |
| 21 | 39 | 45 | 13 | 21 |  |
| 35 | 35 | 46 | 78 | 96 |  |
| 95 | 35 | 31 | 21 | 67 |  |
| 79 | 35 | 76 | 63 | 78 |  |
| 33 | 90 | 62 | 80 | 68 |  |
| 91 | 26 | 48 | 32 | 77 |  |
| 46 | 49 | 25 | 23 | 74 |  |
| 80 | 88 | 50 | 92 | 90 |  |
| 32 | 94 | 77 | 45 | 90 |  |
| 66 | 86 | 67 | 79 | 44 |  |
| 35 | 71 | 80 | 79 | 37 |  |
| 30 | 49 | 59 | 66 | 97 |  |
| 76 | 42 | 85 | 97 | 20 |  |
| 78 | 82 | 90 | 70 | 12 |  |
| 18 | 61 | 70 | 84 | 27 |  |
| 14 | 91 | 64 | 17 | 64 |  |
| 79 | 17 | 68 | 92 | 48 |  |
| 53 | 95 | 29 | 59 | 78 |  |
| 18 | 89 | 76 | 55 | 46 |  |
| 76 | 87 | 89 | 69 | 26 |  |
| 72 | 85 | 93 | 46 | 58 |  |
| 49 | 41 | 93 | 70 | 86 |  |
| 68 | 86 | 30 | 84 | 21 |  |
| 51 | 50 | 29 | 89 | 47 |  |
| 97 | 41 | 52 | 53 | 16 |  |
| 34 | 63 | 86 | 36 | 43 |  |
| 28 | 58 | 56 | 98 | 46 |  |
| 66 | 68 | 96 | 16 | 23 |  |
| 82 | 59 | 43 | 51 | 55 |  |
| 77 | 61 | 78 | 39 | 46 |  |
| 69 | 36 | 41 | 58 | 61 |  |
| 90 | 45 | 27 | 90 | 86 |  |
| 83 | 36 | 63 | 40 | 55 |  |
| 13 | 27 | 84 | 63 | 96 |  |
| 34 | 10 | 24 | 25 | 56 |  |
| 75 | 74 | 57 | 45 | 74 |  |

**Part 3**

For the set of letters below, construct as many words as possible within 5 minutes. Rewards are increasing in the length of the word, with the Nth letter in a word worth N points. That is while a two letter word is worth 1+2=3 points, a three letter word is worth 1+2+3=6 points, a four letter word is worth 1+2+3+4=10 points, a five letter word is worth 1+2+3+4+5 = 15 points and so on (note: 1 letter words will not count). Each point is worth 0.02EUR, so a two letter word is worth 0.06EUR, a three letter word is worth 0.12EUR, a four letter word is worth 0.20EUR, a five letter word is worth 0.30EUR¸ a six letter word is worth 0.42EUR, a seven letter word is worth 0.56EUR, an eight letter word is worth 0.72EUR, and a nine letter word is worth 0.90EUR. Note each letter can be used only once in one particular word. It means if you already have used an “A” in a word, you cannot use it again in this word. However, you can use it again in the next word.

Letters (in alphabetical order) are:

ADEHINRSTU

Please write down the words you constructed:

**________________________________________________________________________________________________________________________________________________________________________________________________________________________________________________________________________________________________________________________________________________________________________________________________________________________________________________________________________________________________________________________________________________________________________________________________________________________________________________________________________________________________________________________________________________________________________________________________________________________________________________________________________________________________________________________________________________________________________**

**Post-Questionnaire**

Please describe briefly how you made your decisions in this experiment: ________________________________________________________________________________________________________________________________________________________________________________________________________________________________________________________________________________________________________________________

Gender:

- Male
- Female

Age: _______

Major: ______________________

What is your native language? _________________

On a scale from 0 to 10 how much did you enjoy the math task (1-not at all, 10-very much)? ____

On a scale from 0 to 10 how much did you enjoy the words task (1-not at all, 10-very much)? ____
